# Alcalase–Flavourzyme Red Seaweed Hydrolysates as Antioxidants to Enhance Oxidative Stability of DHA Nanoemulsions

**DOI:** 10.3390/foods15111950

**Published:** 2026-06-01

**Authors:** Sakhi Ghelichi, Behdad Shokrollahi Yancheshmeh, Seyed Hossein Helalat, Charlotte Jacobsen

**Affiliations:** 1 National Food Institute, Technical University of Denmark, 2800 Kongens Lyngby, Denmark; beshyan@food.dtu.dk; 2 Cell Death and Metabolism, Center for Autophagy, Recycling and Disease (CARD), Danish Cancer Institute, 2100 Copenhagen, Denmark; seyedh@cancer.dk

**Keywords:** red seaweed, protein hydrolysate, antioxidant activity, interfacial localization, omega-3 delivery systems, lipid oxidation

## Abstract

This study evaluated *Palmaria palmata* hydrolysates produced using Alcalase^®^ and Flavourzyme^®^ at 1, 2, 3, 4, 5, and 10% (*w*/*w* biomass protein, corresponding to AF1-10), and their performance in oil-in-water nanoemulsions under iron-induced oxidation. AF4 showed significantly higher total amino acid and phenolic contents and the strongest Fe^2+^-chelating activity (IC_50_ = 1.64 ± 0.22 mg/mL, *p* < 0.05) and was therefore selected for nanoemulsion stabilization. Nanoemulsions exhibited high physical stability with no significant changes in droplet sizes (D_3,2_ ~77–79 nm), ζ-potential (~−18 to −19 mV), and viscosity (~1.2–1.5 cP) (*p* > 0.05). Dynamic interfacial tension measurements and confocal laser scanning microscopy (CLSM) indicated limited interfacial activity of AF4, with most components remaining in aqueous phase. Compared to the control, AF4 significantly reduced peroxide formation (~100–164 vs. 289–357 meq/kg at Day 4–8, *p* < 0.05) and partially preserved tocopherols. It also delayed the formation of some volatiles during intermediate stages of storage. However, it was less effective than ethylenediaminetetraacetic acid (EDTA). Increasing the AF4 concentration did not further improve oxidative stability. These findings suggest that antioxidant efficacy depends on composition, interfacial behavior, and spatial distribution. Antioxidants with limited interfacial activity may therefore exhibit different modes of action within emulsified systems.

## 1. Introduction

Docosahexaenoic acid (DHA; 22:6n-3), a long-chain omega-3 fatty acid, offers numerous health benefits supporting brain and visual development, reducing inflammation, enhancing immune and metabolic function, and lowering the risk of chronic conditions such as cardiovascular disease and metabolic syndrome [1]. Although plant-based foods provide omega-3 fatty acids in the form of α-linolenic acid (ALA; 18:3n-3), the human body has a limited capacity to convert ALA into DHA, making plant sources alone insufficient to meet physiological DHA requirements [2]. Fish and krill oils have traditionally been the major dietary source of DHA, but their use is limited by several factors, including unsuitability for vegan and vegetarian populations, potential contamination concerns, and growing sustainability and environmental issues associated with overfishing [3]. In contrast, algal oil is a sustainable, vegan, and scalable source of DHA with consistent composition, favorable sensory properties, and minimal contamination, making it an attractive alternative to fish oil for functional food and nutraceutical applications [4].

Despite its nutritional importance, DHA is highly susceptible to lipid oxidation due to its multiple double bonds [5]. Therefore, stabilization strategies are needed to preserve its quality and functionality in food and supplement formulations [6]. Nanoemulsions are increasingly explored as delivery systems for DHA due to their enhanced resistance to aggregation and creaming, along with improved digestibility and absorption [7]. However, the large interfacial area associated with small droplet sizes renders DHA-loaded nanoemulsions highly susceptible to oxidative degradation during storage [8]. Although emulsifiers can reduce droplet aggregation, additional antioxidants are often required to improve the oxidative stability and shelf life of omega-3 nanoemulsions [9,10]. While synthetic antioxidants have been widely used, growing health concerns have increased interest in naturally derived alternatives for improving oxidative stability [11,12].

Seaweed has emerged as a promising source of multifunctional food ingredients owing to their rich composition of bioactive compounds with functional and health-promoting properties [13]. Seaweed-derived proteins [14], peptides [15,16], carbohydrates [17,18], and phenolic compounds [19,20] have been shown to exhibit a wide range of functional and biological activities. Proteins and peptides have gained particular attention due to their ability to act as natural antioxidants when released through enzymatic hydrolysis [21]. Among red seaweeds, *Palmaria palmata* has attracted growing interest because of its wide availability and suitability for food applications [22]. Its use in food products is permitted under European Union regulations, supporting its potential for commercial utilization [23]. Previous investigations have shown that protein-rich extracts derived from *P. palmata* using enzymatic [24,25], chemical [26,27], or combined [28,29] extraction approaches exhibit antioxidant activity in vitro. Nonetheless, the functionality of these extracts within complex food matrices has not been extensively established, highlighting the need to evaluate their performance in practical systems such as omega-3-enriched emulsified foods.

Our previous studies demonstrated that enzymatic treatment followed by alkaline extraction produced seaweed extracts capable of enhancing the oxidative stability of omega-3 oil-in-water emulsions [7]. To eliminate the use of chemical solvents and adopt greener processing approaches, the present study investigates the use of purely enzymatic hydrolysis to obtain bioactive extracts with the potential to improve the oxidative stability and shelf life of omega-3-enriched emulsions. Alcalase^®^ and Flavourzyme^®^ were chosen due to their complementary proteolytic activities and their previously demonstrated effectiveness in improving protein recovery and antioxidant potential in *P. palmata* [21]. Earlier work showed that the simultaneous application of these proteases yielded hydrolysates with high protein content and recovery when each enzyme was applied at 1% of the biomass protein content (dry weight basis) [21]. Building on these findings, this study examines the effect of increased enzyme concentrations (2, 3, 4, and 5% of protein content) on hydrolysate protein yield and antioxidant activity, while 10% is also included as a substantially higher enzyme level. This allows for evaluation of whether increasing enzyme addition beyond the intermediate concentration range could further enhance hydrolysis efficiency and antioxidant-related properties, or whether saturation effects would limit additional improvements. The main objective of this study is to evaluate the potential of *P. palmata* protein hydrolysates as natural antioxidants for improving the oxidative stability of algal DHA oil-in-water nanoemulsions. Therefore, the optimal enzymatic treatment, selected based on in vitro free radical scavenging and metal ion chelating capacities, is subsequently applied to stabilize algal DHA oil-in-water nanoemulsions at different hydrolysate concentrations and is compared with nanoemulsions without added antioxidants (negative control) and those containing EDTA as a positive control.

## 2. Materials and Methods

### 2.1. Red Seaweed Biomass

*P. palmata* was collected in the Faroe Islands in early 2024 and purchased from a commercial supplier (Dansk Tang, Nykøbing Sj., Denmark). The biomass was freeze-dried (ScanVac CoolSafe, LaboGene A/S, Allerød, Denmark) to reduce moisture content and then milled using a KN 295 Knifetec™ laboratory mill (Foss A/S, Hillerød, Denmark) to obtain particles of approximately 0.5–1.0 cm. The resulting milled biomass was stored in sealed plastic bags at −20 °C and kept away from light prior to further processing.

### 2.2. Enzymes, DHA Oil, and Chemicals

All enzyme-assisted steps employed two commercial proteolytic preparations: Alcalase^®^ (2.4 amino acid units, AU-A/g) and Flavourzyme^®^ (500–1000 leucine aminopeptidase units, LAPU/g), which were kindly supplied by Novenesis A/S (formerly Novozymes A/S, Bagsværd, Denmark). DHA oil used in this study was a microalgal oil from *Schizochytrium* sp. (Polaris, Quimper, France) containing 57.0 ± 0.3% DHA, measured through fatty acid methyl ester (FAME) analysis by gas chromatography (GC) as explained in [30]. In addition, α-, β-, γ-, and δ-tocopherols (measured as described in Section 2.6.6) were present at 133 ± 0.9, 20 ± 0.3, 697 ± 2.9, and 302 ± 6.6 μg/g, respectively. HPLC-grade solvents were obtained from Lab-Scan (Dublin, Ireland). Amino acid standards were bought from Sigma-Aldrich (St. Louis, MO, USA), and disodium EDTA, 1,1-diphenyl-2-picrylhydrazyl (DPPH) radical, and butylated hydroxytoluene (BHT) were obtained from Sigma-Aldrich (Steinheim, Germany). Unless stated otherwise, other reagents including ammonium bicarbonate, sodium acetate, imidazole, Tween^®^ 20, sodium hydroxide, sodium azide (NaN_3_), tocopherol standards, ammonium thiocyanate, barium dichloride (BaCl_2_·2H_2_O), iron chloride (FeCl_3_·6H_2_O), 96% ethanol, hydrogen peroxide, and volatile standards were acquired from Sigma-Aldrich, while the remaining chemicals were obtained from Merck (Darmstadt, Germany). Ultrapure water was produced at DTU Food using a Milli-Q^®^ Advantage A10 deionization system (Millipore Corporation, Billerica, MA, USA).

### 2.3. Preparation of Red Seaweed Proteolytic Hydrolysates

For enzymatic hydrolysis, dried biomass powder (4 g) was dispersed in deionized water (80 mL) to obtain a 1:20 (*w*/*v*) suspension and allowed to hydrate in a water bath at 50 °C for 1 h. The natural pH of the suspension was within the optimal range for enzyme activity (approximately 6–8); therefore, no chemical pH adjustment was required. Prior to hydrolysis, the protein content of the dried biomass was determined using the Dumas combustion method (Section 2.4.1) and found to be 12.38 ± 1.34% (dry weight basis). Hydrolysis was carried out using a combination of Alcalase^®^ and Flavourzyme^®^, with each enzyme applied at concentrations of 1, 2, 3, 4, 5, and 10% relative to the protein content of the biomass (dry weight basis). The samples were labeled as AF1, AF2, AF3, AF4, AF5, and AF10 corresponding to these enzyme concentrations. The reaction mixtures were incubated in a shaking water bath at 50 °C and 80 rpm for 24 h [21]. The pH was measured before and after hydrolysis, and no significant changes were observed. Enzyme inactivation was carried out by heating the reaction mixtures at 95 °C for 15 min. Following hydrolysis, the samples were centrifuged at 4400× *g* for 15 min at 4 °C (Thermo Scientific™, Sorvall Lynx 4000, Waltham, MA, USA), after which the supernatants were separated and further clarified by passing through a sieve with an approximate mesh size of 1 mm. Both liquid hydrolysates and solid residues were subjected to sequential freezing at −20 °C for 2 h and −80 °C for 6 h prior to freeze-drying (LaboGene A/S, Allerød, Denmark). The resulting dried materials were sealed in zip-lock bags and stored at −80 °C until further analysis. Sample masses were recorded using an analytical balance with a readability of 0.01 g to support mass balance calculations. Non-hydrolyzed protein control was not included, as the study focused on the properties of enzymatic hydrolysates.

### 2.4. Characterization of Hydrolysates

#### 2.4.1. Protein Content, Protein Recovery, and Degree of Hydrolysis (DH)

The protein content of the enzymatic extracts was determined indirectly by measuring total nitrogen using the Dumas combustion method with a Vario EL Cube analyzer (Elementar Analysensysteme GmbH, Langenselbold, Germany). Protein levels were then estimated by applying a nitrogen-to-protein conversion factor of 5.0, which has been reported to be more accurate than 6.25 because of the non-protein nitrogen content in seaweed [31]. The efficiency of protein extraction from the biomass was calculated as protein recovery using the following equation:

(1) $$Protein\;recovery\left(\%\right)=\frac{M_{EH}\times P_{EH}}{M_{RSB}\times P_{RSB}}\times 100$$

where $M_{EH}$ and $P_{EH}$ represent the mass and protein content of the enzymatic hydrolysate, and $M_{RSB}$ and $P_{RSB}$ correspond to the mass and protein content of the red seaweed biomass, respectively.

DH in the enzymatic extracts was assessed using the o-phthaldialdehyde (OPA) assay. Although seaweed extracts may contain non-protein components, OPA is highly selective toward primary amino groups released during protein hydrolysis and has limited reactivity toward other functional groups [32]. Therefore, the assay was considered suitable for relative comparison of DH among hydrolysates under identical experimental conditions. The OPA reagent was prepared following the procedure described in [21]. Extracts were diluted to protein concentrations between 0.05% and 0.25% and combined with the OPA reagent in a microplate format. Absorbance was recorded at 340 nm, and the hydrolysate concentrations were quantified using a calibration curve constructed with L-serine. Serine equivalents were then calculated according to the following equation:

(2) $$Serin\;eequivalents\left(mg\;Ser/mL\right)=\;\frac{\left(A_{E}-\;A_{B}\right)-intercept}{Slope}\;\times dilution\;factor$$

where $A_{E}$ and $A_{B}$ represent hydrolysate and blank absorbances, respectively, and the slope and intercept were derived from the L-serine standard curve. DH was subsequently calculated using the following equation:

(3) $$DH\left(\%\right)=\;\frac{E}{P\;\times 10}\;\times 100$$

where E denotes the serine equivalents of hydrolysate (mg Ser/mL) and P is % protein content. DH measurements were carried out in duplicate.

#### 2.4.2. Amino Acid Composition

Approximately 30 mg of each sample was hydrolyzed in 6 M HCl at 110 °C for 18 h. After filtration (0.22 µm), aliquots were diluted with KOH and ammonium acetate buffer (pH 3.1), yielding a total dilution factor of 32. Amino acid composition was determined by LC-MS (Agilent 1260 Infinity II coupled to a 6120 Quadrupole MS, ESI, Agilent Technologies, Santa Clara, CA, USA) using a BioZen Glycan column (Phenomenex, Torrance, CA, USA) under gradient elution, as previously described [21]. Quantification was performed with external standards (17 amino acids). During hydrolysis, glutamine and asparagine were converted to their corresponding acids, while tryptophan and cysteine were not detected due to degradation.

#### 2.4.3. Total Phenolic Content (TPC)

TPC of the hydrolysates was determined using the Folin–Ciocalteu colorimetric assay [33]. Prior to analysis, hydrolysates were dissolved in distilled water at a concentration of 1 mg/mL. Hydrolysate solutions were then combined with the Folin–Ciocalteu reagent and allowed to react at room temperature for 5 min, after which 6% (*w*/*v*) sodium bicarbonate solution was added. The resulting mixtures were incubated for 60 min under ambient conditions prior to measuring absorbance at 725 nm using a UV-visible spectrophotometer (Shimadzu UV mini 1240, Duisburg, Germany). Phenolic content was quantified as micrograms of gallic acid equivalents (µg GAE) per milliliter of extract, based on a gallic acid standard curve, and calculated using the following equation:

(4) $$TPC\;\left(µg\;GAE/mL\;extract\right)=\;\frac{\left(A-S\right)}{I}$$

where A represents the absorbance of the sample, and S (0.007) and I (0.021) correspond to the slope and intercept of the gallic acid calibration curve, respectively. It should be noted that this assay is based on electron-transfer reactions and may respond not only to phenolic compounds but also to other reducing substances, including amino acids present in protein hydrolysates. Therefore, the reported TPC values should be interpreted with care and not considered an exclusive measure of phenolic compounds [34].

#### 2.4.4. In Vitro Free Radical Scavenging and Metal Ion Chelating

DPPH radical scavenging activity was evaluated using a microplate-based adaptation of the method described in [35], employing a multi-plate reader (Eon^TM^ spectrophotometer, BioTek Instruments, Winooski, VT, USA). Hydrolysates were dissolved in distilled water at various concentrations, and aliquots (100 µL) were mixed with an equal volume of 0.1 mM DPPH solution prepared in ethanol. The reaction mixtures were incubated in the dark at room temperature for 30 min, after which absorbance was measured at 517 nm. Distilled water was used as the blank, while control samples contained extract mixed with 95% ethanol in place of the DPPH solution. All measurements were performed in triplicate, and BHT (0.2 mg/mL) served as the positive control. The percentage of DPPH radical scavenging activity was calculated using the following equation:

$$DPPH\;radical\;scavenging\;(\%)=\left(1-\frac{\left(A_{Hyd}-A_{Ctrl}\right)}{A_{Bnk}}\right)\times 100$$

where $A_{Hyd}$, $A_{Ctrl}$, $A_{Bnk}$ and denote the absorbance values of hydrolysate, control, and blank, respectively. The IC_50_ value, defined as the extract concentration required to inhibit 50% of DPPH radicals, was determined from linear regression analysis of concentration-response curves.

The ferrous ion chelating activity of the extracts was measured following a modified version of the method reported in [36], tailored for microplate measurements. Briefly, 100 µL of extract solution was combined with 110 µL of distilled water and 20 µL of 2 mM FeCl_2_. After a 3 min reaction period, 20 µL of 5 mM ferrozine was added to initiate complex formation. The mixtures were gently mixed and allowed to stand at room temperature for 10 min before absorbance was recorded at 562 nm. Distilled water was used as the blank, while control samples were prepared without the addition of Fe^2+^ and ferrozine. All measurements were conducted in triplicate, with EDTA (0.06 mM) included as a positive control. Ferrous ion chelating activity was calculated using the equation below:

(5) $${Fe}^{2+}chelating\;(\%)=\left(1-\frac{\left(A_{H}-A_{C}\right)}{A_{B}}\right)\times 100$$

where $A_{H}$, $A_{C}$ and $A_{B}$ denote the absorbances of hydrolysate, control, and blank, respectively. IC_50_ values were calculated by linear regression of dose–response data and represent the extract concentration required to chelate 50% of ferrous ions.

#### 2.4.5. Dynamic Interfacial Tension (IFT)

Interfacial activity of the selected hydrolysate for stabilization of nanoemulsions was characterized through dynamic measurements of oil–water interfacial tension. The hydrolysate was dissolved in 10 mM sodium acetate–imidazole buffer (pH 7) at three concentrations (0.82, 1.64, and 3.28 mg/mL) consistent with those employed in the nanoemulsion experiments. Dynamic interfacial tension measurements were conducted by forming pendant drops of medium-chain triglyceride (MCT) oil, which were recorded at 2 s intervals for 30 min using an automated optical tensiometer (OCA25, DataPhysics Instruments GmbH, Filderstadt, Germany) maintained at 25 °C. Interfacial tension values were obtained by fitting drop shapes to the Young–Laplace model, and the evolution of IFT over time was used to assess interfacial adsorption behavior. Measurements performed with buffer alone and Milli-Q water served as reference systems.

### 2.5. Preparation of Nanoemulsions

Nanoemulsions were prepared to evaluate the antioxidant potential of the hydrolysate showing the highest in vitro antioxidant activity, considering both radical scavenging and metal chelating capacities. The hydrolysate was tested at three concentrations based on its iron chelating activity IC_50_: 0.82 mg/mL (half IC_50_), 1.64 mg/mL (IC_50_), and 3.28 mg/mL (twice IC_50_). Solutions of the hydrolysate were prepared in 10 mM sodium acetate-10 mM imidazole buffer (pH 7). EDTA (0.025 mg/mL) was used as a positive control, and a formulation without any antioxidant served as the negative control. Nanoemulsions (220 g) were formulated with 5 wt% DHA oil and 1 wt% Tween^®^ 20. Pre-emulsification was performed using a high-shear mixer (Ultraturrax, Ystral, Germany) at 16,000 rpm for 3 min, during which the oil phase was gradually added to the aqueous hydrolysate solution. The emulsions were then homogenized with a microfluidizer (M110L, Microfluidics, Newton, MA, USA) equipped with a ceramic interaction chamber (CIXC, F20Y, 75 µm) at 9000 psi for three passes to produce a stable nanoemulsion. Sodium azide (0.05 wt%) and FeSO_4_ (50 µM) were included as a preservative and an accelerator of iron-mediated oxidation, respectively. The nanoemulsions were labeled as follows: Em-Ctrl (negative control, no antioxidant), Em-EDTA (positive control, EDTA), Em-AF4a (hydrolysate at 0.82 mg/mL, half IC_50_), Em-AF4b (hydrolysate at 1.64 mg/mL, IC_50_), and Em-AF4c (hydrolysate at 3.28 mg/mL, twice IC_50_). All nanoemulsions were stored in the dark at room temperature (19–20 °C) for up to 8 days. Samples for antioxidant and lipid oxidation analyses were collected on days 0, 2, 4, 6, and 8, transferred into amber vials, flushed with nitrogen to minimize oxidation, and stored at −40 °C until further use.

### 2.6. Characterization of Nanoemulsions

#### 2.6.1. Droplet Size

Nanoemulsion droplet size was characterized on days 1 and 8 using a laser diffraction analyzer (Mastersizer 2000, Malvern Instruments Ltd., Worcestershire, UK) as previously described [37]. Size distribution was expressed as the surface-area mean diameter (D_3,2_) and the volume-weighted mean diameter (D_4,3_). Each determination was carried out in duplicate.

#### 2.6.2. Zeta Potential

Zeta potential measurements of nanoemulsion droplets were performed on the second day using a Zetasizer Nano ZS (Malvern Instruments Ltd., Worcestershire, UK) as previously described [37].

#### 2.6.3. Viscosity

Apparent viscosity was measured using a stress-controlled rheometer (Stresstech, Reologica Instruments AB, Lund, Sweden) fitted with a CC25 bob-and-cup geometry. Tests were performed at 25 °C under a shear stress range of 0.0025–2 Pa. Viscosity readings were taken at a shear rate of 100 s^−1^ and expressed in centipoise (cP).

#### 2.6.4. Confocal Laser Scanning Microscopy (CLSM)

CLSM was employed to visualize the microstructure of nanoemulsions. Samples were prepared with minimal handling to maintain droplet integrity and prevent aggregation. Lipid and protein phases were simultaneously stained using Nile Red and Nile Blue, respectively, by adding both dyes to the sample and incubating for 5 min at room temperature. Nile Red selectively labeled neutral lipid-rich droplets, while Nile Blue targeted protein-containing structures. To avoid structural disturbance, no washing steps were performed after staining. Imaging was carried out using a Zeiss LSM 900 Airyscan microscope (ZEISS, Oberkochen, Germany) equipped with ZEN Blue software (version 2.6) and a 60× oil-immersion objective, as described before [37].

#### 2.6.5. Peroxide Value (PV)

Lipids were extracted from nanoemulsions in duplicate using a modified Bligh and Dyer protocol [38], reduced the volume of chloroform/methanol (1:1, *w*/*w*) required. The PV of the lipid extracts was then quantified in duplicate using the ferrous thiocyanate colorimetric method, with absorbance measured at 500 nm. Unlike the original Shantha and Decker procedure [39], which employs cumene hydroperoxide for calibration and reports PV as mmol peroxide/kg lipid, the present study utilized FeCl_3_ to construct the standard curve and expressed PV as meq peroxide/kg lipid [40]. This approach was selected to enable consistent quantification and relative comparison of peroxide formation among treatments within the system studied. Therefore, absolute PVs should be compared with caution to studies using different calibration standards or calculation methods.

#### 2.6.6. Tocopherol Depletion

Tocopherol levels in lipid extracts were determined using normal-phase high-performance liquid chromatography (HPLC) in accordance with AOCS Official Method Ce 8-89 [41]. Analyses were carried out on an Agilent 1100 series HPLC system (Agilent Technologies, Santa Clara, CA, USA) under isocratic conditions with a mobile phase of heptane and 2-propanol (100:0.4, *v*/*v*) at a flow rate of 1.0 mL/min. A 20 µL sample was injected onto a Waters Spherisorb silica column (Waters Corporation, Milford, MA, USA; 3 µm, 4.6 mm × 150 mm) equipped with a guard column (5 µm, 4.6 mm × 10 mm). Fluorescence detection was performed at 290 nm (excitation) and 330 nm (emission). Prior to injection, approximately 2 g of each extract was dissolved in 1 mL of heptane. Quantification of individual tocopherols was based on external standards using single-point calibration. All measurements were duplicated, and the results are expressed as micrograms of tocopherol per gram of oil.

#### 2.6.7. Volatile Secondary Oxidation Compounds

Volatile secondary oxidation products were analyzed using an automated dynamic headspace sampler (Gerstel GmbH & Co. KG, Mülheim an der Ruhr, Germany) coupled to a GC–MS system (Agilent 6890 N/5973, Agilent Technologies, Santa Clara, CA, USA). Approximately 1 g of sample was spiked with 4-methyl-1-pentanol (30 µg/g) as an internal standard, mixed thoroughly, and incubated at 60 °C for 4 min with intermittent agitation. Volatile compounds were purged with nitrogen (50 mL/min for 20 min), trapped on Tenax GR 300 sorbent tubes (Supelco, Merck KGaA, St. Louis, MO, USA), dried, and thermally desorbed into the GC. Separation was performed on a DB-1701 capillary column (30 m × 0.25 mm ID, 0.5 µm film) using a programmed temperature gradient from 35 °C to 240 °C. Detection was carried out by mass spectrometry in electron ionization mode (70 eV), scanning *m*/*z* 30–250. Compound identification was based on the Wiley 138K spectral library, and quantification employed six-point calibration curves (1–250 µg/mL) prepared from authentic standards under identical conditions. Target volatiles included 4-methyl-1-pentanol (internal standard), 1-penten-3-one, 1-penten-3-ol, hexanal, and (*E,E*)-2,4-heptadienal. Each sample was analyzed in triplicate.

### 2.7. Statistical Analysis

Data analysis was conducted using one-way Analysis of Variance (ANOVA) to test for differences among treatments, followed by Bonferroni’s post hoc adjustment for pairwise comparisons. Changes in droplet size between Day 1 and Day 8 within each emulsion were assessed using paired t-tests. All statistical computations were performed in OriginPro 2023 (OriginLab Corporation, Northampton, MA, USA), and significance was established at *p* < 0.05.

## 3. Results and Discussion

### 3.1. Characteristics of Red Seaweed Hydrolysates

#### 3.1.1. Protein Content, Protein Yield, and DH

Table 1 summarizes the protein content, protein yield, and DH of enzymatic hydrolysates obtained from *P. palmata* using combined Alcalase^®^ and Flavourzyme^®^ treatments at varying enzyme concentrations. DH ranged from approximately 61% to 73% across all treatments, with no significant differences observed among conditions (*p* > 0.05). This indicates that even the lowest enzyme dosage (1%, *w*/*w* relative to protein content) was sufficient to achieve extensive hydrolysis under the applied conditions [42]. The pH of the hydrolysis mixtures was measured before and after the treatment, and no considerable changes were observed, indicating that the reaction conditions remained within the effective activity range of the enzymes during hydrolysis. The absence of a dose-dependent increase suggests that further enzyme addition did not substantially affect hydrolysis extent, likely due to substrate limitation, restricted accessibility of protein within the algal matrix, and enzyme saturation effects [31]. This behavior may also be attributed to the complementary characteristics of the enzyme system used. Alcalase^®^, a serine endoprotease with broad substrate specificity, cleaves internal peptide bonds and likely contributed substantially to the high DH observed [43]. In contrast, Flavourzyme^®^, which possesses predominantly exopeptidase activity together with some endopeptidase activity, mainly hydrolyzes terminal peptide bonds and further processes peptides into smaller fragments and amino acids [44]. Consequently, extensive hydrolysis could be achieved even at relatively low enzyme concentrations, limiting the effect of increasing enzyme dosage.

A comparable trend was observed for protein content and protein yield. Protein content was lowest in AF2 (~9%), which was significantly lower than all other treatments (*p* < 0.05), including AF1 despite its lower enzyme concentration. However, increasing enzyme dosage beyond AF1 did not lead to higher protein content (*p* > 0.05). Protein yield showed a similar pattern, with AF4 presenting the highest value (~51%), although it was not significantly different from AF3, AF5, and AF10 (*p* > 0.05), while AF2 remained significantly lower than AF4 (*p* < 0.05). Overall, variations in enzyme concentration did not translate into proportional changes in either protein content or yield. The comparatively lower protein content and yield observed for AF2 may reflect variability in the recovery of soluble hydrolysis products associated with matrix-related interactions and extraction efficiency rather than differences in hydrolysis extent, since DH values remained comparable among treatments.

These results suggest that extraction efficiency was governed less by enzyme dosage and more by structural and physicochemical constraints inherent to the algal matrix. Limited enzyme accessibility due to polysaccharide–protein associations, diffusion constraints, and restricted exposure of hydrolysable sites likely reduced the impact of increasing enzyme levels [21]. In addition, interactions between released peptides and other cellular components such as polysaccharides and phenolic compounds may have reduced solubility and hindered recovery in the soluble fraction [45]. Furthermore, as discussed earlier, the complementary activities of Alcalase^®^ and Flavourzyme^®^ may have promoted rapid initial protein breakdown and peptide release [43], while matrix-related constraints limited further improvements in hydrolysis efficiency at higher enzyme concentrations. Together, these factors appear to dominate over enzyme concentration effects once a sufficient level of hydrolysis is reached [31].

#### 3.1.2. TPC

The highest and lowest TPC were observed in AF4 (9.07 ± 0.39 µg GAE/mL) and AF2 (6.79 ± 0.25 µg GAE/mL), respectively (Table 1), both of which differed significantly from the other treatments (*p* < 0.05). No significant differences were detected among the remaining hydrolysates (*p* > 0.05), indicating that enzyme concentration had a limited and non-linear effect on TPC. The increase in TPC observed in AF4 may be attributed to enhanced release of phenolic compounds from the algal matrix during enzymatic hydrolysis. Proteolytic enzymes can disrupt protein–phenolic interactions and cell-wall structures, thereby facilitating the liberation of bound phenolics into the soluble fraction [46]. However, because the Folin–Ciocalteu assay may also respond to reducing peptides and amino acids generated during hydrolysis, the reported TPC trends should be interpreted with care and may reflect the combined contribution of phenolic and non-phenolic reducing compounds present in the hydrolysates [34]. Conversely, the lower TPC observed in AF2 suggests that under certain conditions, phenolic compounds may remain bound to macromolecules or become involved in insoluble complexes, limiting their extractability [47]. The lack of a clear dose–response relationship across treatments further indicates that increasing enzyme concentration does not necessarily promote additional phenolic release. Similar observations have been reported for *P. palmata*, where increasing enzyme dosage did not enhance and in some cases even reduced phenolic recovery [21]. This may be due to competing phenomena such as the formation of peptide–phenolic complexes or oxidative reactions that reduce measurable phenolic content [47]. Additionally, the structural complexity of red seaweed cell walls, rich in polysaccharides, can influence the accessibility and release of phenolic compounds during hydrolysis [48]. Overall, these results suggest that moderate enzyme concentrations may favor the release of phenolic compounds, while higher or suboptimal conditions do not necessarily enhance TPC and may even limit phenolic recovery due to interaction or stability constraints.

#### 3.1.3. Amino Acid Composition

Table 2 presents the amino acid composition of red seaweed hydrolysates produced using different concentrations of Alcalase^®^ and Flavourzyme^®^. A clear pattern emerges in which AF4 consistently exhibits significantly higher concentrations of most amino acids (*p* < 0.05) compared with all other hydrolysates. This trend is particularly evident for hydrophobic amino acids (phenylalanine, leucine, isoleucine, and valine), several non-essential amino acids (proline, threonine, and serine), and basic amino acids (arginine and lysine). Accordingly, both total amino acids (TAA) and essential amino acids (EAA) were highest in AF4, indicating a markedly enhanced release of amino nitrogen components under this condition. Interestingly, the magnitude of the differences in amino acid levels does not parallel the protein content results, where AF4 did not differ significantly from AF1, AF5, or AF10 (*p* > 0.05) (Table 1). Although AF4 showed higher protein content than AF2 and AF3 (*p* < 0.05), the relative increase in TAA is substantially greater than the corresponding increase in total protein. This difference may indicate that total nitrogen-based protein estimates and amino acid-derived measurements may respond differently to matrix effects and recovery efficiency, particularly in complex algal hydrolysates.

AF4 yielded the highest absolute EAA content, which was significantly higher than all other hydrolysates (*p* < 0.05), except AF10 (*p* > 0.05). In contrast, AF10 exhibited the highest EAA/TAA ratio (0.40), followed by AF1 and AF4, indicating a relatively greater proportion of essential amino acids under these conditions. These variations likely reflect differences in enzyme specificity and cleavage patterns at different enzyme concentrations [31], as discussed previously for the distinct but complementary catalytic activities of Alcalase^®^ and Flavourzyme^®^ [43]. Among individual amino acids, several residues associated with antioxidant activity were present at significantly higher levels in AF4 compared to the other hydrolysates (*p* < 0.05). Hydrophobic and aromatic amino acids such as phenylalanine, leucine, isoleucine, tyrosine, and valine were markedly elevated in AF4, which is relevant given their known role in enhancing radical scavenging and lipid-phase interactions [49]. Basic amino acids such as arginine, which can participate in electron transfer and metal ion interactions [50], were likewise increased. It should be noted, however, that sulfur-containing amino acids may have been underestimated due to cysteine degradation during acid hydrolysis, which should be considered when interpreting the amino acid profile and its potential relationship to antioxidant activity. The elevated levels of these functionally relevant amino acids suggest that AF4 contains peptide fractions with enhanced antioxidant potential, particularly in systems where hydrophobicity and radical stabilization play a key role. Overall, these results demonstrate that enzyme concentration exerts a pronounced but non-linear effect on amino acid release and distribution. The consistently elevated amino acid levels observed in AF4, together with its high TAA and EAA contents, indicate that this condition promotes extensive hydrolysis and efficient recovery of soluble amino acid fractions. The discrepancy between protein content and amino acid composition further highlights that total protein measurements alone are insufficient to describe hydrolysis efficiency or nutritional quality in enzymatically treated seaweed systems.

#### 3.1.4. In Vitro Antioxidant Activity

**Figure 1:**
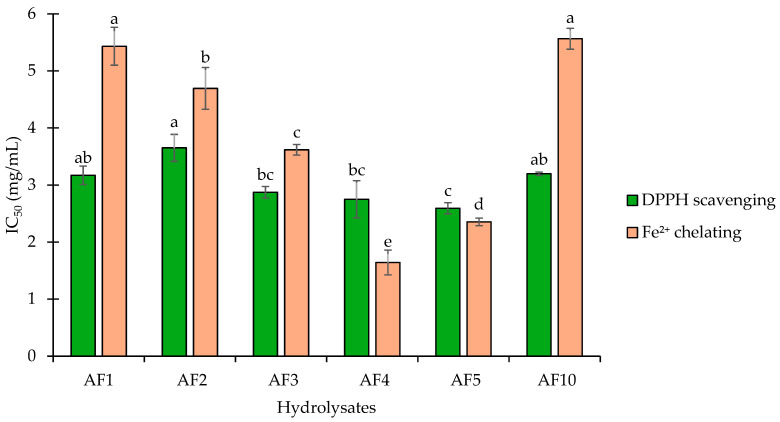
In vitro free radial scavenging and metal ion chelating activities of red seaweed enzymatic hydrolysates. Data are expressed as mean ± standard deviation (*n* = 3). Different superscript letters indicate significant differences among hydrolysates (*p* < 0.05). AF denotes enzymatic hydrolysis using Alcalase^®^ and Flavourzyme^®^, and the number indicates the enzyme concentration (% *w*/*w* relative to protein content of the biomass, dry weight basis). BHT (0.2 mg/mL) and EDTA (0.06 mM) were used as positive controls for DPPH radical scavenging and Fe^2+^ chelating assays, showing 79.7% and 77.0% activity, respectively.

The in vitro antioxidant activity of the hydrolysates is presented in Figure 1 as IC_50_ values for DPPH radical scavenging and Fe^2+^ chelating capacity. For DPPH scavenging, the IC_50_ values ranged from approximately 2.6 to 3.6 mg/mL, indicating a relatively narrow variation in radical scavenging capacity among the different enzymatic treatments. AF5 exhibited the lowest IC_50_ value (2.59 ± 0.10 mg/mL), corresponding to the highest radical scavenging activity, followed by AF4 (2.75 ± 0.33 mg/mL) and AF3 (2.87 ± 0.10 mg/mL). In contrast, AF2 showed the highest IC_50_ value (3.65 ± 0.24 mg/mL), indicating comparatively lower antioxidant activity, while AF1 (3.17 ± 0.09 mg/mL) and AF10 (3.20 ± 0.03 mg/mL) exhibited intermediate values. Despite these numerical differences, the relatively narrow range of IC_50_ values suggests that enzymatic hydrolysis conditions had only a moderate influence on DPPH radical scavenging capacity. This limited variability indicates that differences in enzyme concentration did not translate into substantial changes in antioxidant performance in this assay. The comparatively lower activity of AF2 may be explained by its significantly lower protein content and TPC (*p* < 0.05) (Table 1), which likely reduced the availability of antioxidant-active compounds. Both phenolic compounds and specific amino acid residues are known to contribute to radical scavenging through hydrogen donation and radical stabilization mechanisms [9]. Therefore, the observed DPPH scavenging activity likely reflects the combined contribution of peptides, amino acids, and other reducing components present in the hydrolysates rather than phenolic compounds alone. However, the lack of a strong differentiation among the remaining hydrolysates suggests that DPPH scavenging activity is governed not only by the total concentration of such compounds but also by the structural characteristics of peptides generated during hydrolysis. In particular, peptide size, sequence, and the accessibility of functional amino acid residues play a key role in determining radical scavenging efficiency [51].

The Fe^2+^ chelating activity of the hydrolysates, expressed as IC_50_ values, showed a markedly wider variation compared to DPPH radical scavenging (Figure 1), indicating a stronger influence of enzymatic hydrolysis on this antioxidant mechanism. The IC_50_ values ranged from circa 1.6 to 5.6 mg/mL. AF4 exhibited the lowest IC_50_ value (1.64 ± 0.22 mg/mL), indicating the highest metal chelating activity, followed by AF5 (2.35 ± 0.07 mg/mL) and AF3 (3.62 ± 0.09 mg/mL). In contrast, AF1 (5.43 ± 0.33 mg/mL) and AF10 (5.57 ± 0.18 mg/mL) showed the highest IC_50_ values, corresponding to the lowest chelating capacities, while AF2 (4.69 ± 0.37 mg/mL) exhibited intermediate activity. The superior chelating activity observed in AF4 compared with other hydrolysates may be partially attributed to its higher content of amino acids and peptides capable of binding metal ions (Table 2). Amino acids such as histidine, aspartic acid, glutamic acid, and cysteine are known to play a key role in metal chelation due to the presence of functional groups (e.g., carboxyl, amino, and imidazole groups) that can bind to metal ions [49]. In addition, AF4 showed a significantly higher TPC compared to all other hydrolysates (*p* < 0.05) (Table 1), which may have further enhanced its metal-binding ability, as phenolic compounds can also participate in metal ion chelation through their hydroxyl groups [37]. However, the contribution of phenolic compounds should be interpreted with care, as discussed earlier, since the Folin–Ciocalteu assay may also respond to other reducing compounds present in the hydrolysates. Moreover, the comparison with AF10 indicates that composition alone does not fully explain the observed differences. Despite a relatively favorable amino acid profile and comparable phenolic content, AF10 exhibited substantially lower chelating activity. This suggests that peptide structure, including sequence, molecular size, and the accessibility of functional groups, could also play an important role in metal ion binding [52], potentially contributing to the lower chelating activity observed in AF10 despite its relatively favorable composition. Therefore, the enhanced performance of AF4 is likely the result of a combination of favorable amino acid composition, higher phenolic content, and the generation of peptide structures that are more effective in coordinating metal ions. These findings suggest that AF4 combines more promising performance across multiple antioxidant mechanisms, with particularly enhanced effectiveness in metal chelation. Given that both radical scavenging and metal chelation contribute to oxidative stability in complex systems, AF4 was considered the most suitable hydrolysate for subsequent application as a natural antioxidant in nanoemulsion stabilization.

#### 3.1.5. IFT

**Figure 2:**
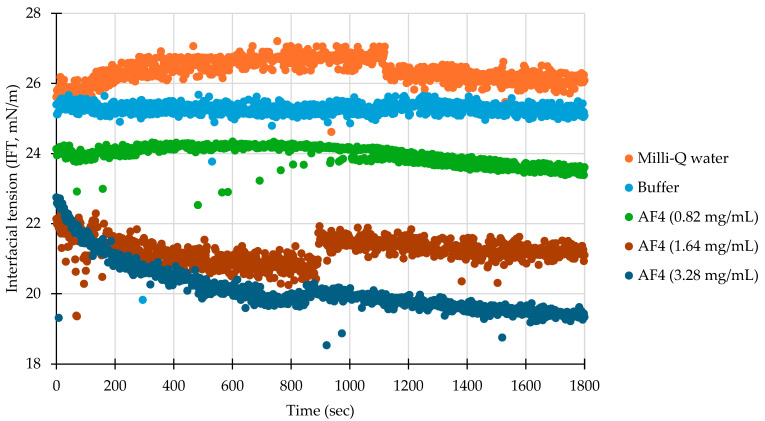
Dynamic interfacial tension (mN/m) between MCT oil and Milli-Q water, sodium acetate-imidazole buffer, and the hydrolysates at three different concentrations. AF denotes enzymatic hydrolysis using Alcalase^®^ and Flavourzyme^®^, and the number indicates the enzyme concentration (% *w*/*w* relative to protein content of the biomass, dry weight basis).

The dynamic interfacial tension between MCT oil and the aqueous phases is presented in Figure 2 for Milli-Q water, buffer, and AF4 hydrolysates at three concentrations. As expected, the IFT values for Milli-Q water and buffer remained relatively constant over time at approximately 25–27 mN/m, confirming the absence of surface-active components in these systems. In contrast, the presence of AF4 hydrolysate resulted in a reduction in IFT, indicating some degree of interfacial activity. However, this reduction was relatively moderate and showed limited dependence on concentration. At 0.82 mg/mL, the IFT stabilized at approximately 24 mN/m, while at 1.64 mg/mL and 3.28 mg/mL, the IFT decreased further to around 21–22 mN/m and 19–22 mN/m, respectively. The decrease in IFT occurred gradually over time, suggesting relatively slow adsorption kinetics compared to highly surface-active molecules. The observed behavior may indicate that the hydrolysate contains components with some amphiphilic character, possibly including peptides generated during enzymatic hydrolysis, which may partially adsorb at the oil–water interface. These peptides may possess both hydrophobic and hydrophilic domains, which may enable partial alignment at the interface and potentially contribute to IFT reduction [37]. However, the relatively high final IFT values and the modest concentration-dependent effect suggest that the interfacial activity of AF4 is limited compared to conventional surfactants. This interpretation seem to be consistent with the compositional characteristics of AF4 discussed previously. Although the hydrolysate showed relatively favorable content of amino acids and bioactive peptides (Table 2), these molecules may not possess sufficient amphiphilicity, structural flexibility, or molecular size distribution required for efficient and dense packing at the interface [53]. Additionally, it is possible that the presence of phenolic compounds and other co-extracted components may influence interfacial behavior through interactions with peptides [54], potentially limiting their ability to orient optimally at the interface. Overall, the results suggest that AF4 could exhibit moderate interfacial activity but may not be able to act as a strong emulsifier on its own. Instead, its primary functionality is probably associated with its antioxidant properties rather than interfacial stabilization. This distinction is important when interpreting its role in nanoemulsion systems, where it is expected to act in the continuous phase rather than competing with the emulsifier at the interface.

### 3.2. Physical Stability of Nanoemulsions

The physical properties of the nanoemulsions, including ζ-potential, droplet size (D_3,2_ and D_4,3_), and viscosity, are presented in Table 3 for Day 1 and Day 8. Overall, all formulations exhibited similar characteristics, indicating that the incorporation of the AF4 hydrolysate at different concentrations did not adversely affect the physical stability of the nanoemulsion system. The ζ-potential values of all nanoemulsions ranged from −18.35 to −19.45 mV on Day 1, with no significant differences observed among treatments (*p* > 0.05). This suggests that the addition of AF4, regardless of concentration, did not significantly alter the surface charge of the oil droplets. The relatively consistent negative charge across all samples is likely governed by the interfacial properties of the emulsifier rather than the added hydrolysate. Although the emulsifier used is nonionic, the presence of negatively charged impurities or ionizable components in the oil phase can impart a measurable surface charge [7]. The magnitude of ζ-potential observed here indicates moderate electrostatic repulsion, which, in combination with steric stabilization provided by the emulsifier layer, contributes to maintaining dispersion stability [37].

The droplet size results further support the physical stability of nanoemulsions. On Day 1, D_3,2_ values ranged from 77.65 to 79.45 nm, while D_4,3_ values ranged from 199.0 to 214.5 nm, with no significant differences observed among the formulations (*p* > 0.05). This indicates that the incorporation of AF4, regardless of concentration, did not influence the initial droplet size distribution. After 8 days of storage, both D_3,2_ and D_4,3_ values remained largely unchanged across all samples, confirming the absence of significant droplet growth, flocculation, or coalescence between the analyzed time points during storage. The stability of droplet size over time demonstrates that the presence of AF4, even at twice the IC_50_ concentration, did not disrupt the interfacial layer or induce instability phenomena. The similarity in droplet size distributions among Em-Ctrl, Em-EDTA, and AF4-containing nanoemulsions suggests that the hydrolysate did not compete effectively with the emulsifier for adsorption at the oil–water interface. This implies that AF4 likely remained in the continuous phase rather than acting as a dominant surface-active component, which is consistent with the dynamic interfacial tension results showing only moderate interfacial activity (Figure 2). Such behavior aligns with the compositional characteristics of the hydrolysate (Table 1), where antioxidant functionality is mainly attributed to soluble peptides and phenolic compounds rather than strong interfacial activity.

The apparent viscosity of the nanoemulsions ranged from 1.18 to 1.42 cP on Day 1 and remained within a similar range after 8 days, with no significant differences among treatments (*p* > 0.05). These values are characteristic of dilute nanoemulsion systems and indicate that the addition of AF4 did not alter the flow behavior of the emulsions. The absence of viscosity changes over time further supports the lack of droplet aggregation or network formation during storage [37]. Overall, the combination of moderate negative ζ-potential, small and stable droplet sizes, and low viscosity demonstrates that all nanoemulsions were physically stable throughout the storage period. Importantly, the incorporation of AF4 at increasing concentrations did not compromise these properties, indicating its compatibility with the nanoemulsion system. This stability is essential for evaluating the functional performance of the hydrolysate in subsequent oxidative stability studies, as it ensures that any observed effects can be attributed to its antioxidant activity rather than changes in nanoemulsion structure.

### 3.3. Microstructure of Nanoemulsions

**Figure 3:**
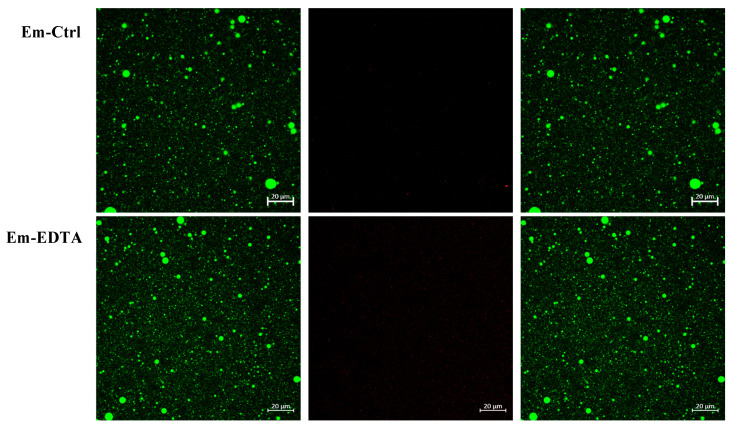

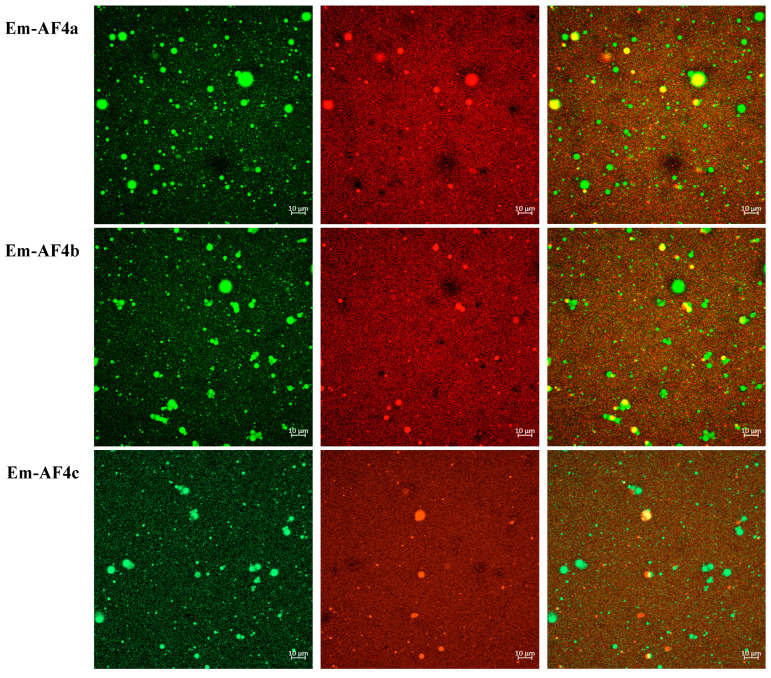
CLSM of DHA nanoemulsions. Oil droplets were stained with Nile Red (green) and AF4 hydrolysate with Nile Blue (red). Panels show oil only (**left**), hydrolysate only ((**middle**); absent in Em-Ctrl and Em-EDTA), and combined staining (**right**). Em-Ctrl (negative control, no antioxidant), Em-EDTA (positive control, EDTA), Em-AF4a (hydrolysate at 0.82 mg/mL, half IC_50_), Em-AF4b (hydrolysate at 1.64 mg/mL, IC_50_), and Em-AF4c (hydrolysate at 3.28 mg/mL, twice IC_50_).

The microstructure of the nanoemulsions was examined by CLSM (Figure 3). Across all formulations, the nanoemulsions exhibited a relatively uniform dispersion of droplets with no clear signs of flocculation or coalescence, which is consistent with the droplet size and ζ-potential results (Table 3). Em-Ctrl and Em-EDTA showed only green fluorescence corresponding to the lipid phase and the droplets appeared well distributed, confirming that the base formulation produced stable nanoemulsions. In contrast, the emulsions containing AF4 (Em-AF4a-c) exhibited increasing red fluorescence intensity with rising hydrolysate concentration. However, unlike systems where strong interfacial absorption occurs, the red fluorescence in these samples was not confined exclusively to the droplet interface. Instead, a substantial portion of the signal appeared distributed throughout the continuous phase, with only partial and diffuse association around some droplets. Even at the highest concentration (Em-AF4c), where the overall fluorescence intensity was greatest, the interfacial localization remained relatively weak and heterogeneous. This distribution pattern suggests that the AF4 hydrolysate components were not strongly surface-active and did not form a dense, continuous interfacial layer. Rather, they were predominantly present in the aqueous phase, with limited adsorption at the oil–water interface. This observation aligns with the dynamic interfacial tension results (Figure 2), which showed only moderate reductions in IFT, as well as the droplet size data, where no significant differences were observed among formulations (*p* > 0.05) (Table 3). Together, these findings indicate that AF4 does not effectively compete with the primary emulsifier for interfacial adsorption. The limited interfacial localization of AF4 can be attributed to its compositional characteristics. Although the hydrolysate contains peptides and phenolic compounds with potential amphiphilic properties, these molecules may lack the structural features necessary for strong and stable interfacial anchoring. Additionally, interactions among peptides and phenolics in the aqueous phase may further reduce their availability for adsorption at the interface. Overall, CLSM analysis supports the conclusion that AF4 does not act as a primary interfacial stabilizer in the nanoemulsion system. Instead, its distribution in the continuous phase suggests that its functional role is more likely associated with bulk-phase antioxidant activity. However, even limited interfacial presence may still contribute to oxidative stability in a dynamic system, where mobile Fe^2+^ ions can diffuse toward or interact with the interface. Importantly, despite this limited interfacial activity, the presence of AF4 did not disrupt droplet organization or physical stability, reinforcing its suitability as a functional additive in nanoemulsion systems.

### 3.4. Oxidative Stability of Nanoemulsion

#### 3.4.1. PV

Primary lipid oxidation in the nanoemulsions was assessed by monitoring PV over 8 days under Fe^2+^-induced pro-oxidant conditions (Table 4). At Day 0, Em-Ctrl already exhibited a high PV (~99 meq/kg oil), indicating rapid hydroperoxide formation during emulsification in the absence of antioxidant protection. This behavior is consistent with the high susceptibility of emulsified lipids to oxidation, particularly in systems containing transition metals and large interfacial areas [55]. During storage, oxidation in Em-Ctrl progressed rapidly, with PV increasing sharply to ~357 meq/kg oil by Day 8, remaining significantly higher than all other formulations (*p* < 0.05). This pronounced instability aligns with the rapid depletion of endogenous antioxidants and may suggest that no effective protective mechanism was present in this system. In contrast, Em-EDTA maintained consistently low PVs (~16–34 meq/kg oil) throughout storage, reflecting the strong inhibitory effect of metal chelation on oxidation. This result highlights the dominant role of iron-catalyzed reactions in this model system and provides a benchmark for evaluating the effectiveness of the hydrolysate. Nanoemulsions containing AF4 exhibited intermediate oxidative stability, with significantly lower PVs than the control (*p* < 0.05) but higher values than Em-EDTA. This could indicate that AF4 provided measurable antioxidant protection under pro-oxidant conditions. It is noteworthy that during storage, peroxide values in some samples were already close to or above the upper sensitivity limits of the ferric thiocyanate assay from the beginning, and in others approached this limit at later stages. Therefore, while absolute values at these points should be interpreted with caution, the observed trends remain reliable for comparing oxidative progression among treatments. The absence of a clear linear dose–response relationship, particularly the lack of further improvement at the highest concentration (Em-AF4c), could suggest that increasing AF4 concentration in the nanoemulsion does not necessarily translate into enhanced oxidative stability. Although AF4 was previously characterized by high TAA and elevated levels of antioxidant-related residues (Table 2), and had the highest TPC (Table 1), these compositional advantages did not result in proportional improvements at higher concentrations. This observation suggests that antioxidant efficacy in emulsified systems is governed not only by composition but also by the localization and mode of action of active compounds [9]. AF4 exhibited significantly stronger Fe^2+^ chelating activity compared to other hydrolysates (*p* < 0.05) (Figure 1), indicating its potential to interfere with metal-catalyzed oxidation. Since Fe^2+^ ions are primarily present in the aqueous phase, this chelating ability could contribute to reducing radical generation by binding pro-oxidant metal ions. However, compared to EDTA, the strength of these interactions is presumably lower, allowing residual iron to remain catalytically active. As a result, initiation reactions may not be fully suppressed, and oxidation can still proceed. Moreover, lipid oxidation in emulsions predominantly propagates at the oil–water interface, where lipid substrates are concentrated [55]. In this regard, dynamic interfacial tension measurements (Figure 2) showed that AF4 possesses only moderate surface activity, while CLSM observations (Figure 3) suggested that its components are mainly distributed in the continuous phase, with limited accumulation at the droplet interface. Thus, the proposed bulk-phase and interfacial behavior of AF4 should be considered a plausible interpretation based on indirect evidence rather than a directly confirmed mechanism. Consequently, although AF4 may act in the aqueous phase through partial metal chelation, its limited interfacial localization may partly explain its restricted ability to inhibit propagation reactions occurring at the interface.

#### 3.4.2. Tocopherol Consumption

The evolution of α-, γ-, and δ-tocopherols during storage (Table 4) provides further insight into oxidative processes occurring in the nanoemulsions under Fe^2+^-induced conditions. The initial tocopherol profile reflected that of the oil phase, characterized by relatively high levels of γ- and δ-tocopherols and low α-tocopherol, while β-tocopherol was not detected. Accordingly, the discussion focuses on α-, γ-, and δ-homologues. In Em-Ctrl, tocopherol depletion was rapid and extensive. γ-Tocopherol was not detected at any time point, indicating immediate consumption under severe oxidative stress. α-Tocopherol remained low throughout storage (2.58–3.36 µg/g), while δ-tocopherol decreased markedly from 25.34 µg/g at Day 0 to 10.21 µg/g at Day 8 (*p* < 0.05). These results are consistent with the high PVs and confirm intense radical formation in the absence of effective antioxidants. In contrast, Em-EDTA showed the highest level of tocopherol preservation. α-Tocopherol remained stable (~9–10.5 µg/g), and γ-tocopherol was maintained at high levels (~34–37 µg/g) throughout storage. Although δ-tocopherol decreased after Day 2, its levels (~13–14 µg/g) remained significantly higher than in the control at later stages (*p* < 0.05). This confirms that EDTA effectively inhibited iron-catalyzed oxidation and minimized tocopherol consumption [37]. AF4-containing nanoemulsions (Em-AF4a-c) exhibited intermediate and homolog-dependent tocopherol preservation, reflecting a partial antioxidant effect. Compared to Em-Ctrl, these systems did not consistently show higher tocopherol levels across all homologues and time points; however, they demonstrated clear improvements in specific cases, particularly for γ-tocopherol, which was present at substantial levels in all AF4 systems at Day 0 (~29–34 µg/g), in contrast to its complete absence in Em-Ctrl. Although γ-tocopherol decreased progressively during storage, appreciable amounts remained at Day 8 (~15–19 µg/g), potentially indicating that AF4 might have contributed to delaying its depletion. This behavior suggests that AF4 was effective, at least to some extent, in mitigating oxidative processes affecting γ-tocopherol, showing a trend qualitatively similar to that observed in Em-EDTA, albeit less pronounced. In contrast, α-tocopherol showed rapid depletion in AF4 systems, particularly in Em-AF4b and Em-AF4c, where it reached undetectable levels by Day 4 and 6, respectively. In these cases, α-tocopherol levels were comparable to or lower than those in Em-Ctrl, indicating limited protection. δ-Tocopherol remained relatively stable in AF4 systems (~11–19 µg/g), but initial levels were lower than in Em-Ctrl, and no consistent improvement over the control was observed during storage. The different behavior of α- and γ-tocopherol is consistent with previous studies showing that tocopherol efficiency in emulsions depends not only on radical-scavenging activity, but also on interfacial localization and regeneration mechanisms [55]. In oil-in-water emulsions, less methylated tocopherol homologues can outperform α-tocopherol, whereas α-tocopherol is often more effective in bulk oils, highlighting the importance of the emulsion interface for antioxidant function [56]. Rapid depletion of α-tocopherol in Em-AF4b and Em-AF4c may therefore indicate that α-tocopherol was preferentially consumed during the early stages of oxidation without being efficiently regenerated, since α-tocopherol depletion has been shown to track the onset of hydroperoxide and hexanal formation.

Overall, no clear dose-dependent effect of AF4 concentration was observed (*p* > 0.05), suggesting that increasing hydrolysate levels did not systematically enhance tocopherol preservation. However, the sustained presence of γ-tocopherol and the moderate stability of δ-tocopherol may suggest that AF4 could potentially exert a measurable, though limited, antioxidant effect. These findings can be explained by the physicochemical characteristics of AF4. As previously shown, AF4 contains amino acids, peptides, and phenolic compounds (Table 1 and Table 2) with antioxidant and Fe^2+^-chelating properties (Figure 1). These components apparently contribute to reducing oxidative stress in the aqueous phase. However, their limited interfacial activity (Figure 2) and predominant localization in the continuous phase (Figure 3) restrict their ability to effectively inhibit radical propagation at the oil–water interface. Consequently, endogenous tocopherols, especially α-tocopherol, remain actively involved in scavenging lipid radicals and are progressively depleted. Nevertheless, the partial preservation of γ- and δ-tocopherols suggests that AF4 might have provided a degree of oxidative protection, recommending its potential as a natural antioxidant ingredient, although its efficacy under these conditions remains lower than that of EDTA.

#### 3.4.3. Volatile Secondary Oxidation Products

**Figure 4:**
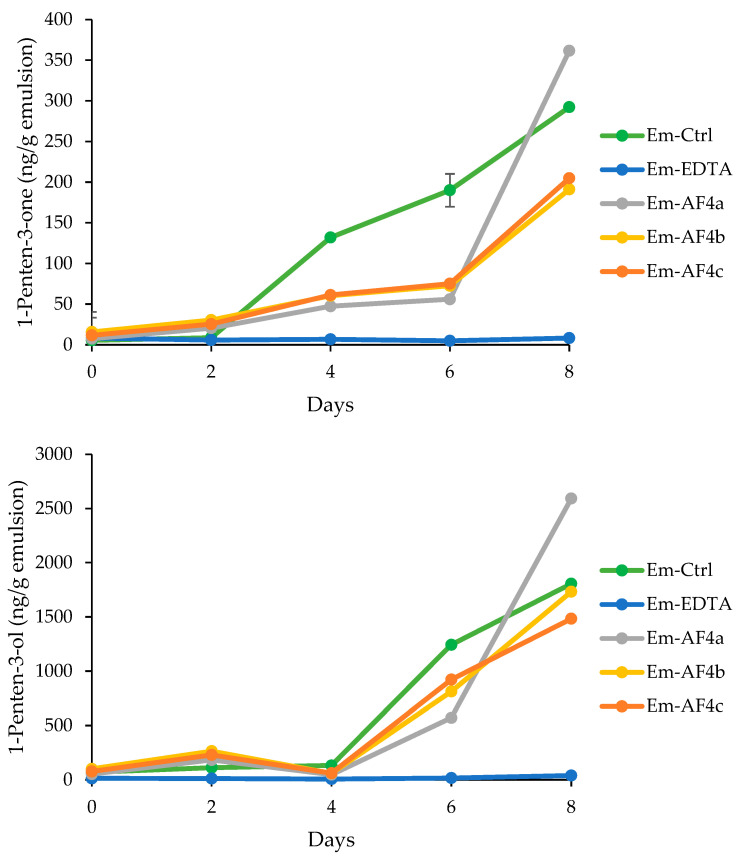

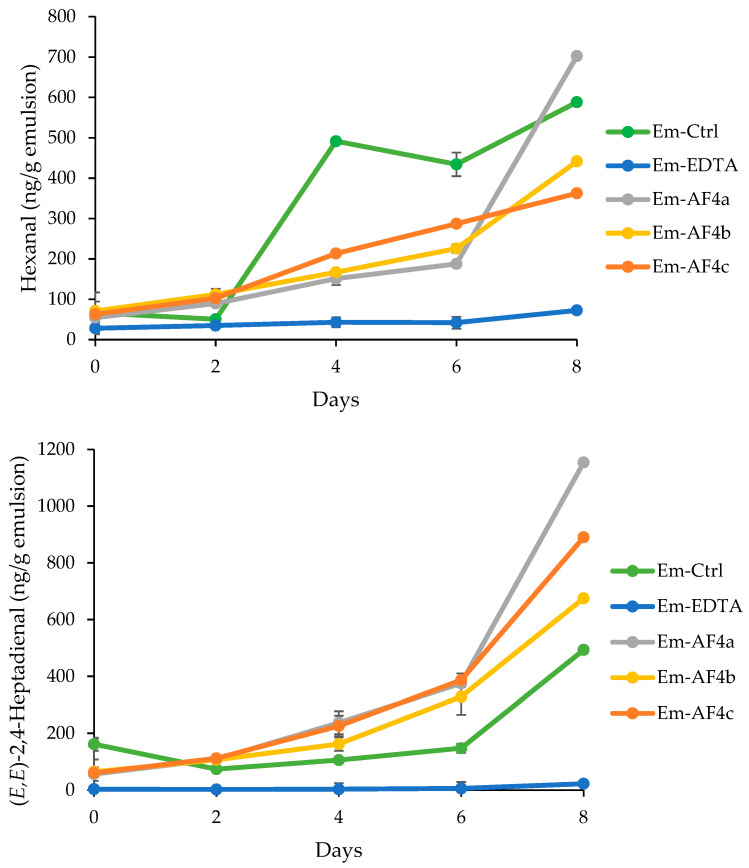
Secondary oxidation volatile compounds of DHA oil-in-water nanoemulsions (*n* = 3) in an 8-day storage: Em-Ctrl (negative control, no antioxidant), Em-EDTA (positive control, EDTA), Em-AF4a (hydrolysate at 0.82 mg/mL, half IC_50_), Em-AF4b (hydrolysate at 1.64 mg/mL, IC_50_), and Em-AF4c (hydrolysate at 3.28 mg/mL, twice IC_50_).

The formation of secondary lipid oxidation products, including 1-penten-3-one, 1-penten-3-ol, hexanal, and (*E,E*)-2,4-heptadienal, was monitored during storage as key indicators of ω-3 PUFA degradation (Figure 4). These compounds originate from the decomposition of lipid hydroperoxides and provide insight into the progression from primary to secondary oxidation [7]. Across all volatiles, Em-Ctrl exhibited the highest accumulation, confirming extensive oxidative deterioration under Fe^2+^-induced conditions. For example, 1-penten-3-ol increased sharply from 68.94 ng/g at Day 0 to 1806.32 ng/g at Day 8 (*p* < 0.05), while 1-penten-3-one rose from 5.59 to 292.32 ng/g over the same period. Similarly, hexanal and (*E,E*)-2,4-heptadienal reached 588.22 ng/g and 493.48 ng/g, respectively, by Day 8. These results are consistent with the high PVs observed in Em-Ctrl and reflect rapid hydroperoxide decomposition into secondary oxidation products [9]. In contrast, Em-EDTA effectively suppressed the formation of all monitored volatiles throughout storage. At Day 8, 1-penten-3-ol and 1-penten-3-one remained at only 39.86 ng/g and 8.32 ng/g, respectively, both significantly lower than in all other systems (*p* < 0.05). Similar trends were observed for hexanal (72.52 ng/g) and (*E,E*)-2,4-heptadienal (22.25 ng/g). These findings confirm that EDTA efficiently inhibits iron-catalyzed oxidation, limiting both hydroperoxide formation and their subsequent breakdown, in agreement with the strong tocopherol preservation observed in this system [37]. AF4-containing nanoemulsions (Em-AF4a-c) exhibited intermediate behavior, with volatile levels generally lower than Em-Ctrl but higher than Em-EDTA, indicating a partial antioxidant effect. During the early stages of storage (Day 2), differences among AF4 systems and the control were relatively small and often not statistically significant (*p* > 0.05), suggesting limited initial protection under strong prooxidant conditions. However, from Day 4 onwards, clearer differences emerged. For 1-penten-3-ol, AF4 systems showed a delayed increase compared to Em-Ctrl. At Day 6, values ranged from 555.77 to 812.26 ng/g in AF4 samples, significantly lower than the control (1243.22 ng/g, *p* < 0.05). A similar trend was observed for 1-penten-3-one, where AF4 samples exhibited values between 55.95 and 75.14 ng/g at Day 6, compared to 190.07 ng/g in Em-Ctrl (*p* < 0.05). These results indicate that AF4 was effective in slowing the decomposition of primary oxidation products into secondary volatiles during the propagation phase. For hexanal, a marker of ω-6 PUFA secondary oxidation pathways, AF4 systems also showed reduced formation compared to the control at intermediate stages. At Day 6, hexanal levels ranged from 166.79 to 225.60 ng/g in AF4 systems, significantly lower than Em-Ctrl (434.09 ng/g, *p* < 0.05). However, variability was relatively high at earlier time points, and differences were not always statistically significant, reflecting the complex formation pathways of aldehydes [55]. The formation of (*E,E*)-2,4-heptadienal, a characteristic oxidation product of polyunsaturated fatty acids, followed a similar pattern. AF4 systems exhibited significantly lower levels than Em-Ctrl at Day 6 (e.g., 328.58–386.69 ng/g vs. 493.48 ng/g, *p* < 0.05), indicating partial inhibition of advanced lipid oxidation. Nevertheless, this compound increased markedly at later stages, particularly in Em-AF4a and Em-AF4c. At Day 8, volatile levels in AF4 systems increased substantially for all compounds. For example, in Em-AF4b, 1-penten-3-ol reached 1732.99 ng/g, while 1-penten-3-one increased to 191.13 ng/g. Comparing these results to those measured for Em-Ctrl indicates that the differences were less pronounced than at earlier stages, indicating a decline in antioxidant effectiveness of AF4 over time. No consistent dose-dependent trend was observed among AF4 concentrations (*p* > 0.05), although moderate reductions were occasionally observed at higher concentrations depending on the compound.

The observed volatile profiles are closely aligned with the PV and tocopherol data (Table 4). The delayed formation of volatiles in AF4 systems during the early and mid-stages of storage corresponds to lower PVs and the partial preservation of γ- and δ-tocopherols. In particular, the sustained presence of γ-tocopherol likely contributed to scavenging lipid radicals and slowing secondary oxidation [57]. However, the rapid depletion of α-tocopherol and the progressive consumption of γ-tocopherol at later stages coincide with the sharp increase in volatile compounds, indicating insufficient long-term protection. From a mechanistic perspective, the moderate antioxidant effect of AF4 appears to be potentially related to its ability to influence the fate of primary oxidation products rather than fully preventing their formation. As shown in PV results (Table 4), AF4-containing systems exhibited some control over hydroperoxide accumulation during the early stages of storage, suggesting a partial inhibition of initiation reactions. This seems consistent with the presence of peptides, amino acids, and phenolic compounds in AF4 (Table 1 and Table 2; Figure 1), which could contribute to radical scavenging and Fe^2+^ chelation in the aqueous phase [37]. However, the volatile data indicates that once hydroperoxides are formed, their subsequent decomposition into secondary oxidation products is not effectively suppressed. The marked increase in compounds such as 1-penten-3-ol and (*E,E*)-2,4-heptadienal at later stages may suggest that AF4 does not sufficiently stabilize hydroperoxides or interrupt propagation pathways. This behavior is in agreement with the progressive depletion of tocopherols, particularly α-tocopherol, which highlights the role of endogenous antioxidants in the defense against radical propagation [55]. This apparent limitation could also be influenced by the structural and colloidal characteristics of the emulsions. While AF4 contributed to maintaining overall emulsion stability, as reflected in the droplet size distribution and ζ-potential (Table 3), these effects do not necessarily translate into effective control of localized oxidation processes. Lipid oxidation is known to be highly site-specific [9], and the accumulation of volatiles suggests that critical reactions at the oil–water interface proceed despite the presence of AF4. Despite these constraints, the consistently lower levels of volatiles in AF4-treated systems compared to the control during the propagation phase could suggest that AF4 may provide a measurable and functionally relevant antioxidant effect. While its efficacy remains lower than that of EDTA, the results may show the potential of AF4 as a natural antioxidant capable of partially mitigating lipid oxidation under prooxidant conditions. Further improvements in performance may be achieved through strategies aimed at enhancing interfacial localization or through synergistic combinations with complementary antioxidants, thereby enabling more effective control of both primary and secondary oxidation processes.

## 4. Conclusions

This study demonstrated that concentration of Alcalase^®^ and Flavourzyme^®^ influenced the compositional and functional properties of *P. palmata* protein hydrolysates, with AF4 generally showing superior biochemical characteristics, including higher amino acid content, phenolic levels, and metal-chelating activity. When applied in nanoemulsions, AF4 showed antioxidant effects by delaying peroxide formation, postponing the formation of some of the volatile oxidation products, especially at intermediate stages of storage, and slowing tocopherol depletion compared to the control. However, its performance remained lower than that of EDTA under Fe^2+^-induced oxidative stress. The results suggest that antioxidant efficacy in emulsified systems may not be solely determined by intrinsic activity, but also by the localization and physicochemical behavior of active compounds. Despite its strong chelating and favorable radical scavenging properties, the limited interfacial activity of AF4 might have restricted its ability to fully inhibit oxidation at the oil–water interface, where lipid oxidation predominantly occurs. However, given that oxidation was iron-catalyzed, its lower efficacy compared to EDTA is more likely related to weaker iron-binding capacity, while interfacial localization may play a secondary role in determining overall antioxidant performance. These findings highlight the importance of considering both molecular composition and spatial distribution when developing natural antioxidants for complex colloidal systems. It should be noted that a non-hydrolyzed protein control was not included in this study, as the primary focus was on the functional properties of enzymatically generated hydrolysates. Future studies could include native protein fractions to better elucidate the specific effects of hydrolysis. Furthermore, future work should focus on enhancing interfacial affinity or combining hydrolysates with complementary antioxidants to achieve more effective oxidative stabilization.

## Figures and Tables

**Table 1:** Protein content (% dry matter), protein yield (%), degree of hydrolysis (DH, %), and total phenolic compounds (TPC, µg GAE/mL) of red seaweed enzymatic hydrolysates.

Treatment	Protein Content (%)	Protein Yield (%)	DH (%)	TPC (µg GAE/mL)
AF1	11.13 ± 0.58 ^ab^	41.41 ± 3.12 ^bc^	67.17 ± 0.85 ^a^	8.00 ± 0.09 ^b^
AF2	9.02 ± 0.08 ^c^	34.71 ± 0.95 ^c^	61.15 ± 9.69 ^a^	6.79 ± 0.25 ^c^
AF3	10.75 ± 0.26 ^b^	43.66 ± 1.67 ^ab^	71.23 ± 1.86 ^a^	7.50 ± 0.74 ^b^
AF4	12.60 ± 0.37 ^a^	50.91 ± 1.84 ^a^	68.26 ± 2.41 ^a^	9.07 ± 0.39 ^a^
AF5	12.47 ± 0.37 ^a^	48.73 ± 0.19 ^ab^	64.88 ± 0.11 ^a^	8.11 ± 0.28 ^b^
AF10	11.75 ± 0.04 ^ab^	47.69 ± 0.17 ^ab^	73.42 ± 0.47 ^a^	8.11 ± 0.08 ^b^

Data are presented as mean ± standard deviation. Protein content and yield were determined in triplicate (*n* = 3), DH in eight replicates (*n* = 8), and TPC in six replicates (*n* = 6). Different superscript letters indicate significant differences among treatments (*p* < 0.05). AF denotes enzymatic hydrolysis using Alcalase^®^ and Flavourzyme^®^, and the number indicates the enzyme concentration (% *w*/*w* relative to protein content of the biomass, dry weight basis).

**Table 2:** Amino acid composition (mg/g dry matter) of red seaweed enzymatic hydrolysates.

	AF1	AF2	AF3	AF4	AF5	AF10
Phenylalanine	4.70 ± 0.09 ^a^	1.24 ± 0.28 ^b^	1.15 ± 0.26 ^b^	5.56 ± 0.16 ^a^	1.48 ± 0.35 ^b^	5.14 ± 0.49 ^a^
Leucine	7.99 ± 0.38 ^c^	1.85 ± 0.08 ^d^	1.79 ± 0.16 ^d^	10.23 ± 0.33 ^a^	7.90 ± 0.50 ^c^	9.01 ± 0.14 ^b^
Isoleucine	4.80 ± 0.23 ^b^	1.25 ± 0.17 ^d^	1.92 ± 0.08 ^c^	6.11 ± 0.13 ^a^	4.27 ± 0.29 ^b^	5.78 ± 0.22 ^a^
Methionine	1.09 ± 0.13 ^b^	1.02 ± 0.08 ^b^	1.02 ± 0.07 ^b^	2.39 ± 0.01 ^a^	1.12 ± 0.04 ^b^	2.46 ± 0.13 ^a^
Tyrosine	3.13 ± 0.30 ^a^	0.69 ± 0.11 ^b^	0.54 ± 0.07 ^b^	3.22 ± 0.01 ^a^	0.96 ± 0.05 ^b^	3.58 ± 0.33 ^a^
Proline	7.17 ± 0.20 ^b^	2.97 ± 0.05 ^d^	2.89 ± 0.05 ^d^	9.10 ± 0.23 ^a^	3.88 ± 0.26 ^c^	7.25 ± 0.19 ^b^
Valine	7.39 ± 0.20 ^b^	2.25 ± 0.39 ^d^	3.55 ± 0.91 ^c^	9.45 ± 0.08 ^a^	7.88 ± 0.24 ^b^	8.55 ± 0.19 ^ab^
Alanine	10.30 ± 0.43 ^b^	13.42 ± 0.34 ^a^	14.47 ± 0.47 ^a^	14.14 ± 0.71 ^a^	14.60 ± 1.04 ^a^	11.45 ± 0.22 ^b^
Threonine	1.92 ± 0.23 ^c^	1.77 ± 0.37 ^c^	1.66 ± 0.23 ^c^	4.03 ± 0.03 ^b^	2.51 ± 0.76 ^c^	5.55 ± 0.08 ^a^
Glycine	7.19 ± 0.26 ^a^	4.09 ± 0.65 ^bc^	2.72 ± 2.13 ^c^	8.94 ± 0.28 ^a^	6.42 ± 0.62 ^ab^	7.83 ± 0.85 ^a^
Serine	3.56 ± 0.77 ^b^	3.22 ± 0.34 ^b^	2.87 ± 0.13 ^b^	6.37 ± 0.27 ^a^	4.35 ± 0.63 ^b^	3.29 ± 0.57 ^b^
Arginine	3.62 ± 0.11 ^c^	2.64 ± 0.08 ^d^	2.51 ± 0.19 ^d^	7.17 ± 0.06 ^b^	3.05 ± 0.24 ^cd^	8.74 ± 0.54 ^a^
Histidine	2.31 ± 0.51 ^a^	2.66 ± 0.04 ^a^	3.25 ± 0.32 ^a^	2.62 ± 0.56 ^a^	2.97 ± 0.19 ^a^	2.49 ± 0.47 ^a^
Lysine	6.78 ± 1.13 ^c^	9.10 ± 0.08 ^ab^	10.46 ± 0.26 ^a^	8.35 ± 0.57 ^abc^	9.38 ± 1.33 ^ab^	7.98 ± 0.35 ^bc^
Glutamic acid	19.19 ± 0.60 ^c^	26.83 ± 0.18 ^ab^	28.88 ± 0.65 ^a^	25.56 ± 0.26 ^b^	28.12 ± 1.91 ^ab^	19.42 ± 0.66 ^c^
Cystine	0.76 ± 0.66 ^a^	1.33 ± 0.69 ^a^	2.01 ± 1.78 ^a^	0.14 ± 0.14 ^a^	2.12 ± 0.22 ^a^	0.20 ± 0.17 ^a^
Aspartic acid	16.85 ± 1.59 ^c^	18.24 ± 0.63 ^bc^	20.20 ± 0.46 ^b^	24.98 ± 0.35 ^a^	25.40 ± 1.75 ^a^	19.36 ± 0.04 ^bc^
TAA	108.77 ± 2.69 ^c^	94.57 ± 0.45 ^d^	101.89 ± 2.70 ^cd^	148.36 ± 4.03 ^a^	126.42 ± 8.83 ^b^	128.07 ± 2.72 ^b^
EAA	40.88 ± 2.49 ^b^	23.16 ± 1.43 ^c^	27.35 ± 1.16 ^c^	52.09 ± 1.99 ^a^	40.59 ± 3.12 ^b^	50.73 ± 1.16 ^a^
EAA/TAA	0.38 ± 0.02 ^ab^	0.24 ± 0.01 ^d^	0.27 ± 0.01 ^d^	0.35 ± 0.00 ^bc^	0.32 ± 0.00 ^c^	0.40 ± 0.00 ^a^

Data are presented as mean ± standard deviation (*n* = 3). Different superscript letters indicate significant differences among treatments (*p* < 0.05). AF denotes enzymatic hydrolysis using Alcalase^®^ and Flavourzyme^®^, and the number indicates the enzyme concentration (% *w*/*w* relative to protein content of the biomass, dry weight basis). TAA and EAA represent total amino acids and essential amino acids, respectively.

**Table 3:** Zeta potential, droplet size, and apparent viscosity of DHA nanoemulsions.

	ζ-Potential (mV) Day 1	D_3,2_ (nm) Day 1	D_3,2_ (nm) Day 8	D_4,3_ (nm) Day 1	D_4,3_ (nm) Day 8	Viscosity (cP) Day 1	Viscosity (cP) Day 8
Em-Ctrl	−18.40 ± 0.57 ^a^	79.35 ± 0.07 ^a^	79.45 ± 0.07 ^a^	206.0 ± 0.00 ^a^	204.0 ± 1.41 ^a^	1.18 ± 0.06 ^a^	1.35 ± 0.08 ^a^
Em-EDTA	−18.95 ± 0.21 ^a^	79.25 ± 0.07 ^a^	78.15 ± 0.07 ^a^	214.5 ± 0.07 ^a^	213.0 ± 0.00 ^a^	1.30 ± 0.00 ^a^	1.43 ± 0.09 ^a^
Em-AF4a	−19.45 ± 0.07 ^a^	78.75 ± 0.00 ^a^	79.15 ± 0.00 ^a^	199.0 ± 0.00 ^a^	198.0 ± 1.41 ^a^	1.40 ± 0.09 ^a^	1.30 ± 0.05 ^a^
Em-AF4b	−19.05 ± 0.49 ^a^	79.45 ± 0.05 ^a^	78.40 ± 0.03 ^a^	209.0 ± 9.90 ^a^	207.0 ± 8.49 ^a^	1.42 ± 0.02 ^a^	1.46 ± 0.07 ^a^
Em-AF4c	−18.35 ± 0.21 ^a^	77.65 ± 0.02 ^a^	77.10 ± 0.00 ^a^	212.5 ± 7.78 ^a^	199.5 ± 0.07 ^a^	1.42 ± 0.05 ^a^	1.46 ± 0.05 ^a^

Data are presented as mean ± standard deviation (*n* = 2). Different superscript letters indicate significant differences among nanoemulsions at each sampling day (*p* < 0.05). Em-Ctrl (negative control, no antioxidant), Em-EDTA (positive control, EDTA), Em-AF4a (hydrolysate at 0.82 mg/mL, half IC_50_), Em-AF4b (hydrolysate at 1.64 mg/mL, IC_50_), and Em-AF4c (hydrolysate at 3.28 mg/mL, twice IC_50_).

**Table 4:** Peroxide value (PV) and tocopherol content of DHA nanoemulsions during an 8-day storage.

Sample	Day	Peroxide Value (meq/kg oil)	α-Tocopherol (µg/g Emulsion)	γ-Tocopherol (µg/g Emulsion)	δ-Tocopherol (µg/g Emulsion)
Em-Ctrl	Day 0	99.43 ± 26.45 ^b,wx^	3.27 ± 0.13 ^a,y^	0.00 ± 0.00 ^a,y^	25.34 ± 1.00 ^a,x^
	Day 2	118.85 ± 4.68 ^b,w^	2.98 ± 0.79 ^a,x^	0.00 ± 0.00 ^a,z^	23.32 ± 1.55 ^a,x^
	Day 4	289.44 ± 8.52 ^a,w^	3.36 ± 0.99 ^a,x^	0.00 ± 0.00 ^a,z^	20.71 ± 0.78 ^ab,w^
	Day 6	336.93 ± 20.14 ^a,w^	2.98 ± 0.13 ^a,x^	0.00 ± 0.00 ^a,y^	17.48 ± 1.11 ^b,wx^
	Day 8	357.15 ± 24.36 ^a,w^	2.58 ± 0.24 ^a,x^	0.00 ± 0.00 ^a,z^	10.21 ± 1.06 ^c,y^
Em-EDTA	Day 0	21.33 ± 0.43 ^b,x^	8.76 ± 0.00 ^b,w^	37.06 ± 0.14 ^a,w^	38.54 ± 0.74 ^a,w^
	Day 2	16.62 ± 1.38 ^b,x^	9.07 ± 0.41 ^ab,w^	36.01 ± 0.21 ^a,w^	39.81 ± 2.78 ^a,w^
	Day 4	22.17 ± 1.59 ^b,y^	10.51 ± 0.22 ^a,w^	35.75 ± 1.35 ^a,w^	14.37 ± 0.47 ^b,x^
	Day 6	34.38 ± 2.15 ^a,y^	10.38 ± 0.02 ^a,w^	34.16 ± 0.55 ^a,w^	13.76 ± 0.17 ^b,x^
	Day 8	32.00 ± 1.00 ^a,x^	10.49 ± 0.52 ^a,w^	34.27 ± 1.77 ^a,w^	13.81 ± 0.65 ^b,wx^
Em-AF4a	Day 0	75.21 ± 1.70 ^a,wx^	4.13 ± 0.66 ^a,xy^	31.82 ± 1.11 ^a,x^	13.07 ± 0.57 ^a,z^
	Day 2	161.04 ± 39.69 ^a,w^	2.52 ± 0.68 ^a,x^	28.56 ± 0.76 ^a,x^	12.70 ± 0.46 ^a,y^
	Day 4	124.08 ± 30.37 ^a,x^	2.95 ± 1.90 ^a,x^	28.44 ± 3.47 ^a,wx^	12.38 ± 0.06 ^a,x^
	Day 6	136.07 ± 37.82 ^a,xy^	1.59 ± 0.24 ^a,y^	26.05 ± 0.60 ^ab,wx^	12.92 ± 0.46 ^a,x^
	Day 8	163.90 ± 37.13 ^a,x^	0.00 ± 0.00 ^a,y^	18.74 ± 1.19 ^b,x^	11.18 ± 0.21 ^a,xy^
Em-AF4b	Day 0	96.24 ± 20.22 ^a,wx^	2.61 ± 0.18 ^a,y^	29.66 ± 0.07 ^a,x^	17.39 ± 0.04 ^a,yz^
	Day 2	89.45 ± 14.74 ^a,wx^	0.66 ± 0.94 ^ab,x^	23.38 ± 0.90 ^ab,y^	16.21 ± 2.50 ^a,xy^
	Day 4	111.73 ± 27.72 ^a,xy^	0.00 ± 0.00 ^b,x^	24.22 ± 1.05 ^ab,xy^	12.08 ± 0.00 ^a,x^
	Day 6	110.62 ± 19.80 ^a,xy^	0.00 ± 0.00 ^b,z^	21.01 ± 3.72 ^b,x^	12.82 ± 0.17 ^a,x^
	Day 8	100.19 ± 21.40 ^a,x^	0.00 ± 0.00 ^b,y^	18.94 ± 0.33 ^b,x^	12.14 ± 0.35 ^a,wxy^
Em-AF4c	Day 0	165.44 ± 47.96 ^a,w^	6.19 ± 0.80 ^a,x^	33.97 ± 3.18 ^a,x^	18.99 ± 1.86 ^a,y^
	Day 2	94.97 ± 20.88 ^a,wx^	3.72 ± 0.01 ^b,x^	29.80 ± 0.03 ^a,y^	18.03 ± 0.02 ^a,xy^
	Day 4	94.76 ± 15.90 ^a,xy^	2.76 ± 0.17 ^b,x^	28.34 ± 2.13 ^a,y^	19.60 ± 1.62 ^a,w^
	Day 6	180.05 ± 13.82 ^a,x^	0.00 ± 0.00 ^c,z^	25.26 ± 2.81 ^ab,x^	18.73 ± 1.97 ^a,w^
	Day 8	158.63 ± 44.69 ^a,x^	0.00 ± 0.00 ^c,y^	15.36 ± 0.61 ^b,y^	14.35 ± 0.27 ^a,w^

Data are presented as mean ± standard deviation (*n* = 2). The superscripts a–c indicate significant differences among the time points for each nanoemulsion, and the superscripts w–z indicate significant differences among different nanoemulsions at each time point (*p* < 0.05). Em-Ctrl (negative control, no antioxidant), Em-EDTA (positive control, EDTA), Em-AF4a (hydrolysate at 0.82 mg/mL, half IC_50_), Em-AF4b (hydrolysate at 1.64 mg/mL, IC_50_), and Em-AF4c (hydrolysate at 3.28 mg/mL, twice IC_50_). Control data were previously reported in an earlier publication [37].

## Data Availability

The original contributions presented in the study are included in the article, further inquiries can be directed to the corresponding authors.
